# Age-associated NF-κB signaling in myofibers alters the satellite cell niche and re-strains muscle stem cell function

**DOI:** 10.18632/aging.101098

**Published:** 2016-11-14

**Authors:** Juhyun Oh, Indranil Sinha, Kah Yong Tan, Bernard Rosner, Jonathan M. Dreyfuss, Ornela Gjata, Peter Tran, Steven E. Shoelson, Amy J. Wagers

**Affiliations:** ^1^ Department of Stem Cell and Regenerative Biology and Harvard Stem Cell Institute, Cambridge, MA 02138; ^2^ Joslin Diabetes Center, Boston, MA 02215, USA; ^3^ Division of Plastic Surgery, Brigham and Women's Hospital, Boston, MA 02115, USA; ^4^ Department of Biostatistics, Harvard School of Public Health, MA 02115, USA; ^5^ Department of Biomedical Engineering, Boston University, Boston 02215, USA; ^6^ Department of Medicine, Harvard Medical School, Boston, MA 02115, USA; ^7^ Paul F. Glenn Center for the Biology of Aging, Harvard Medical School, Boston, MA 02115, USA

**Keywords:** satellite cell, skeletal muscle, aging, regeneration

## Abstract

Skeletal muscle is a highly regenerative tissue, but muscle repair potential is increasingly compromised with advancing age. In this study, we demonstrate that increased NF-κB activity in aged muscle fibers contributes to diminished myogenic potential of their associated satellite cells. We further examine the impact of genetic modulation of NF-κB signaling in muscle satellite cells or myofibers on recovery after damage. These studies reveal that NF-κB activity in differentiated myofibers is sufficient to drive dysfunction of muscle regenerative cells via cell-non-autonomous mechanisms. Inhibition of NF-κB, or its downstream target Phospholipase A2, in myofibers rescued muscle regenerative potential in aged muscle. Moreover, systemic administration of sodium salicylate, an FDA-approved NF-κB inhibitor, decreased inflammatory gene expression and improved repair in aged muscle. Together, these studies identify a unique NF-κB regulated, non-cell autonomous mechanism by which stem cell function is linked to lipid signaling and homeostasis, and provide important new targets to stimulate muscle repair in aged individuals.

## INTRODUCTION

It has long been known that muscle repair potential is increasingly compromised with age [1,2], and that this age-related defect is associated with reduced activity of muscle satellite cells [1,2,3,4] and with the presence of chronic, low grade inflammation in muscle [5]. Working from the hypothesis that a heightened inflammatory tone in aged muscle could contribute to poor regenerative capacity, we developed genetic systems to alter in-flammatory gene expression in satellite cells or muscle fibers by modulating the activity of nuclear factor κB (NF-κB), a master transcriptional regulator of inflammation whose activity is upregulated in many cell types and tissues with age [6,7].

The NF-κB transcription factor plays a central role in multiple systemic and cellular processes [8]. Activation of NF-κB via the classical, or canonical, pathway is triggered by pro-inflammatory signals and involves its release from an inhibitory interaction with cytoplasmic inhibitor of kappa B (IκB) [9]. This release is initiated by a phosphorylation and degradation cascade that activates the IκB kinase β (IKKβ), which in turn phosphorylates IκB and stimulates its ubiquitination and degradation [9]. In the absence of IκB, NF-κB trans-locates to the nucleus where it regulates expression of genes involved in proliferation, differentiation, migration and regulation of the immune and inflammatory systems [10]. NF-κB activation via the alternative pathway similarly requires degradation of IκB, but occurs independent of IKKβ activity.

In skeletal muscle, NF-κB activation has been associated with a variety of degenerative and malignant muscle disorders, including muscular dystrophy, cachexia and rhabdomyosarcoma [10]. Canonical NF-κB signaling is activated in proliferating myoblasts and appears to repress myoblast differentiation, in part by inhibiting expression of the myogenic transcription factor *MyoD* [11,12]. In contrast, the alternative pathway is activated during myoblast fusion to form multinucleated myotubes, where it may regulate mitochondrial biogenesis [13]. Tonic activation of canonical NF-κB signaling in muscle fibers drives progressive muscle atrophy, in part by upregulation of the E3 ubiquitin ligases MURF and MAFbx [14,15]. Conversely, inhibition of NF-κB activity in a variety of cell types, including macrophages and myofibers, can reduce inflammation and fibrosis and accelerate repair after muscle injury [16,17].

Here, we investigate the particular role of canonical NF-κB signaling in the loss of muscle regenerative potential that typically occurs during normal aging. These studies reveal that selective activation of NF-κB activity in muscle fibers drives dysfunction of regenerative muscle satellite cells and that life-long inhibition of NF-κB activity in myofibers preserves muscle repair potential with aging via cell-non-autonomous effects on satellite cell function. Further analysis of differential gene expression in muscles with varying NF-κB activity identified a secreted phospholipase (PLA2G5) as a myofiber-expressed, NF-κB-regulated gene that governs muscle regenerative capacity with age. These data suggest a model in which NF-κB activation in muscle fibers increases PLA2G5 expression and drives the impairment in regenerative function characteristic of aged muscle. Importantly, inhibition of NF-κB function reverses this impairment, suggesting that FDA-approved drugs like salsalate, which diminish NF-κB activity, may provide new therapeutic avenues for elderly patients with reduced capacity to recover effectively from muscle injury.

## RESULTS

### Increased NF-κB activity in myofibers and myotubes, but not in satellite cells alone, impairs satellite cell function

Age-associated deficiencies in muscle repair slow recovery of muscle function and promote replacement of damaged myofibers with fat and fibrous tissue rather than newly formed muscle [2,3]. Based in part on studies in mice and humans suggesting that a pro-inflammatory microenvironment impairs physiological function [14,18,19] and limits repair potential in aged muscle [20], we hypothesized that alterations in canonical NF-κB signaling may underwrite some of the functional changes induced in muscle during aging. Consistent with this hypothesis, muscle satellite cells isolated by fluorescence activated cell sorting (FACS, Fig. S1) from aged (24 months old) mice showed substantially increased expression of many genes that are either direct targets or activators of the NF-κB pathway, including *interleukin 6 (Il-6)*, *Il-33*, *chemokine-chemokine ligand 2* (*Ccl-2*), and *cyclo-oxygenase 2 (Cox-2)*, when compared to young (2-3 months old) mice (Fig. S2A). Similar to previous reports [3,4,21,22,23], satellite cells were reduced in frequency in aged muscle (Fig. S1, S2B) and exhibited substantially impaired myogenic activity when compared to similarly isolated young cells (Fig. S2C). These data thus correlate up-regulation of NF-κB activity in muscle satellite cells with decreased myogenic potential, suggesting that deregulated NF-κB activation may inhibit satellite cell regenerative functions in aged muscle. However, upregulation of NF-κB signaling is not unique to aged satellite cells. Prior studies have reported heightened NF-κB activity in sarcopenic muscle [6,19,24], and electrophoretic mobility shift assays (EMSA) confirmed increased DNA binding activity of NF-κB in muscle fibers from aged mice (Fig. S2D,E). Therefore, to determine if greater NF-κB activity in aged satellite cells themselves might directly impair their regenerative function, we generated a bi-allelic Cre-lox transgenic mouse in which NF-κB can be activated specifically in muscle satellite cells following administration of tamoxifen. This system is similar to that used previously by Guttridge and colleagues to evaluate NF-κB activities in satellite cells during muscle wasting in young, tumor-bearing cachectic mice and during muscle growth in neonatal animals [5].

**Figure 1:**
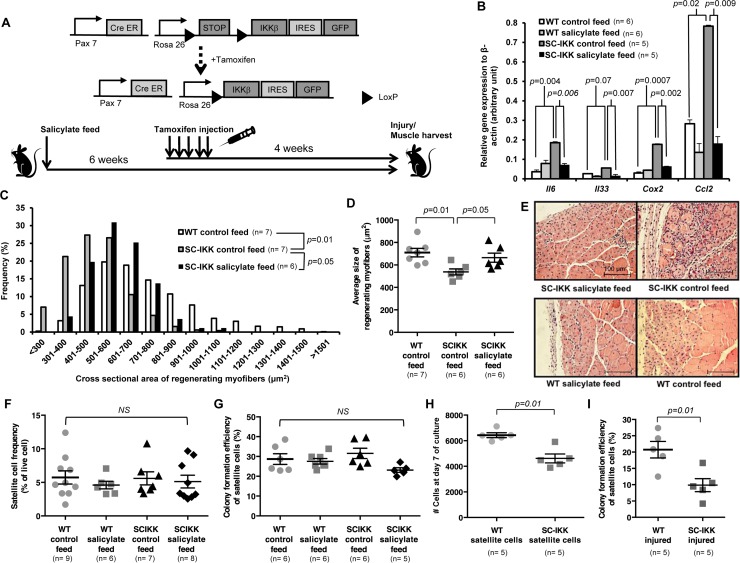
Satellite cell-specific increase of NF-κB activity impairs regenerative function in vivo, but does not affect satellite cell function in vitro (**A**) Biallelic SC-IKK mice contain a satellite cell specific Pax7-CreER allele and a Cre-inducible loxP-flanked STOP sequence with downstream bicistronic IKKβca-IRES-eGFP allele under control of the Rosa26 promoter. Exposure of SC-IKK mice to tamoxifen for 5 consecutive days induces Cre activity in satellite cells and causes satellite cell-specific expression of constitutively active IKKβ, leading to a satellite cell-specific increase in NF-κB activity. All mice received sodium salicylate feed or control feed starting at 2 months of age. Two weeks later, mice were injected with vehicle (corn oil) or tamoxifen, and underwent cryoinjury after an additional 4 weeks. (**B**) Quantification of mRNA levels of NF-κB target gene expression by qRT-PCR in satellite cells isolated from young WT mice receiving control (n=6 mice) or sodium salicylate feed (n=6 mice), or SC-IKK mice receiving control (n=5 mice) or sodium salicylate feed (n=5 mice). P-values calculated by one-way ANOVA. (**C**, **D**) Quantification of regenerating (centrally-nucleated) myofiber size in TA muscles 7 days after cryoinjury for WT mice receiving control feed (n=7 mice) or SC-IKK mice receiving control (n=7 mice) or sodium salicylate feed (n=6 mice). Mice with sodium salicylate treatment continued receiving salicylate feed during recovery after injury. Data presented as histograms of fiber size (binned by 100 μm2 increments, C or as average fiber cross-sectional area (mean ± s.e.m., D). P-values determined by Kruskal-Wallis test with step-down Bonferroni method for (**C**) and (**D**). (**E**) Representative H&E staining of TA muscle sections at 7 days after cryoinjury from tamoxifen-treated SC-IKK and age-matched WT, with or without salicylate feed. Scale bars, 100 μm. (**F**) Flow cytometric analysis of satellite cell frequency in uninjured WT or SC-IKK mice receiving control or salicylate feed (n=6-9 mice per group). (**G**) Frequency of sorted satellite cells from uninjured WT or SC-IKK mice giving rise to myogenic colonies in clonal cell culture (n=5 or 6 mice per group). P-values were calculated by one-way ANOVA and are non-significant for all comparisons in (**F**) and (**G**). (**H**) Number of cells at day 8 of culture started with 1000 satellite cells isolated from tamoxifen-treated SC-IKK or WT mice (n=5 mice per group, 3 technical replicates per mouse). (**I**) Myogenic colony forming efficiency of satellite cells isolated from muscles of tamoxifen-treated SC-IKK or WT mice 5 days after cardiotoxin injury (n=5 mice per group). P-values calculated by Student's t test. Data represent mean ± s.e.m., if not noted otherwise.

Mice carrying the satellite cell-specific tamoxifen-inducible Pax7-CreER transgene [25] were crossed with mice carrying a Cre-inducible bicistronic IKKβca-IRES-eGFP sequence (encoding a constitutively active (ca) form of IKKβ and eGFP) with upstream loxP-flanked STOP cassette driven by the Rosa26 promoter [26] (Fig. 1A). Tamoxifen injection in these bi-allelic “SC-IKK” mice (named for ‘satellite cell’ specific IKKβ production) induced IKKβca-IRES-eGFP expression in the majority of satellite cells, as assessed by GFP expression (Fig. S3). Consequent expression in these cells of constitutively active IKKβca, which ensures continuous degradation of IκB and release of NF-κB from its inhibitory complex [26], potently induced the expression in satellite cells of multiple NF-κB regulated inflammatory genes (Fig. 1B). Importantly, this induction could be rescued by systemic treatment with the NF-κB inhibitor sodium salicylate [27], confirming involvement of NF-κB activation in this perturbation of the satellite cell transcriptome (Fig. 1B).

To assay the impact of constitutive NF-κB activation in satellite cells on muscle regeneration, we challenged tamoxifen-treated SC-IKK mice by cryoinjury, which induces necrotic cell death of a select region of muscle (typically ~20% in our studies), and activates satellite cells in the surrounding tissue to nucleate repair [3,4,28]. Analysis of regenerating myofibers in previously cryodamaged muscles revealed a clear deficit in repair in tamoxifen-treated SC-IKK mice, evidenced by reduced cross-sectional area of newly formed fibers (marked by central nuclei, Fig. 1C-E) at day 7 after injury. This regenerative defect was largely ameliorated by administration of sodium salicylate prior to injury and during muscle repair (Fig. 1C-E), conforming involvement of heightened NF-κB activity in the observed phenotype. Surprisingly, satellite cells isolated from SC-IKK mice prior to injury showed no differences in frequency (Fig. 1F) or in their intrinsic ability to initiate and expand myogenic colonies in single-cell culture (Fig. 1G, Fig. S4, colony number and size scored at day 5 after single cell seeding). However, when satellite cells were seeded at greater numbers (1000 cells per well) and allowed to differentiate for 7-8 days, so that they formed multinucleated myotubes, we found that cultures from uninjured SC-IKK mice contained fewer nuclei as compared to cultures from wildtype (WT) controls (Fig. 1H, Fig. S5). These data suggested that the presence of multinucleated derivatives of SC-IKK satellite cells may have a negative impact on the proliferation or survival of these cells, consistent with potential paracrine signaling among SC-IKK muscle cells. In further support of this notion, the myogenic activity of SC-IKK satellite cells was markedly reduced when these cells were isolated from injured muscle (Fig. 1I). Thus, based on these data, we contemplated the possibility that incorporation of nuclei from NF-κB activated SC-IKK satellite cells into regenerated muscle fibers *in vivo*, or myotubes *in vitro*, creates an NF-κB-activated niche that negatively impacts the further myogenic function of resident muscle satellite cells. In such a model, activation of NF-κB signaling in aged muscle fibers, rather than aged satellite cells, would represent the primary cause of deficient muscle regeneration in older animals.

### Muscle fiber-specific blockade of NF-κB activity improves satellite cell function in aging mice

**Figure 2:**
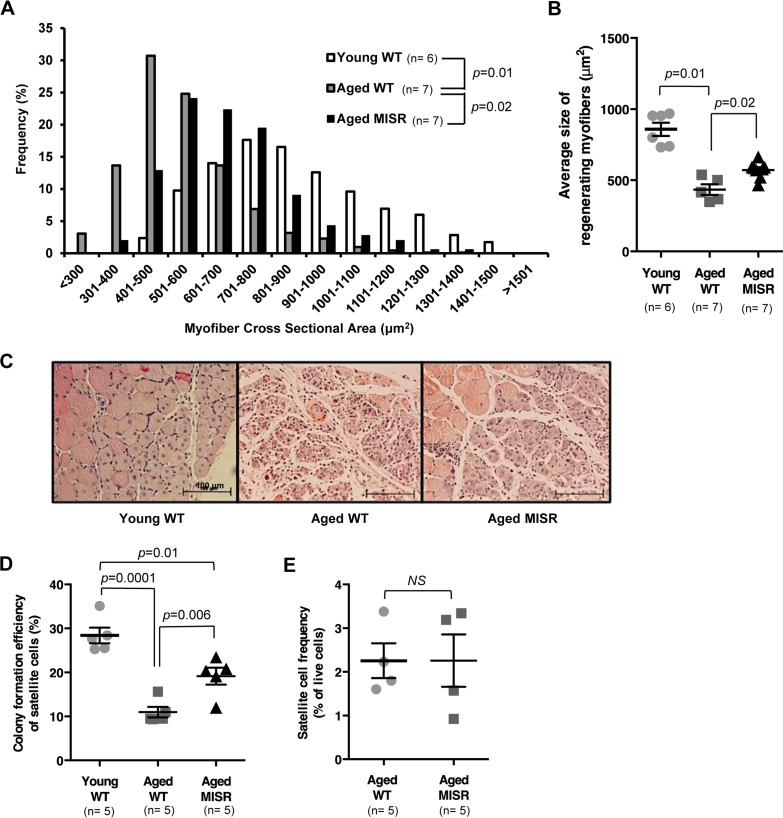
Muscle fiber-specific transgenic inhibition of NF-κB activity improves satellite cell function in an aging model (**A**, **B**) Distribution and average of cross-sectional area of regenerating (centrally nucleated) fibers in injured TA muscle of young (n=6 mice) or aged WT (n=7 mice), or aged MISR mice (n=7 mice) at 7 days after cryoinjury. Data represented as histograms of fiber size (**A**) or as mean ± s.e.m. (**B**). P-values calculated by Kruskal-Wallis test with Step-down Bonferroni method. (**C**) Representative H&E staining of muscle sections taken 7 days after cryoinjury in young WT, aged WT or aged MISR mice. Scale bars, 100 μm. (**D**) Myogenic colony forming efficiency of satellite cells from young (n=5 mice) or aged (n=5 mice) WT or aged MISR (n=5 mice) mice. Data presented as mean ± s.e.m. P-values calculated by one-way ANOVA. (**E**) Frequency of satellite cells (percent of live cells by flow cytometry) in uninjured muscle of aged MISR (n=5 mice) or aged WT mice (n=5 mice). MISR mice were allowed to age alongside age-matched wild-type controls for these studies. Data presented as mean ± s.e.m. P-values calculated by Student's t test. For all studies, young mice were 2-3 months old, and aged mice were 24 months old.

One possible strategy to test the hypothesis that activation of NF-κB in muscle fibers rather than in satellite cells is a critical driver of age-associated defects in muscle regenerative potential would be to use a muscle-specific Cre driver to activate the same IKKβca allele in muscle fibers. In fact, such animals have been generated using the muscle fiber-specific muscle creatine kinase- (MCK-) Cre allele [14] ; however, analysis of skeletal muscle in these “MIKK” animals (for muscle fiber specific IKKβ production) revealed a profound muscle wasting phenotype, which would likely confound any evaluation of satellite cell number or function in these animals. Therefore, to evaluate the importance of muscle fiber-specific activation of NF-κB signaling in muscle aging, we took a converse approach, examining satellite cell and muscle regenerative function in aged “MISR” mice, which harbor a transgene that dominantly blocks NF-κB signaling in skeletal muscle fibers (but not satellite cells) via constitutive expression of the IκB super-repressor (ISR) under the MCK promoter (Fig. S6A). MISR mice exhibit nearly complete inhibition of NF-κB activity in mature myofibers in young [14] and aged (24 month) animals (Fig. S6B,C). Life-long inhibition of NF-κB in MISR mice protected these animals from age-related loss of muscle regenerative potential, as indicated by an increased caliber of regenerating fibers following cryoinjury in aged MISR mice as compared to age-matched wild-type controls (Fig. 2A-C). Improved regeneration in aged MISR mice was accompanied by an improvement in satellite cell myogenic activity (Fig. 2D), although age-related reductions in satellite cell frequency were unaffected (Fig. 2E). No differences were noted in muscle repair or in satellite cell activity or frequency in young MISR mice, compared to littermate controls (Fig. S7). Thus, modulating NF-κB regulated gene expression in mature muscle fibers produces a cell-non-autonomous impact on resident satellite cells, consistent with a local “niche” effect of aging on satellite cell function.

### Decreased pla2g5 expression in myofibers improves muscle regeneration in aged mice

**Figure 3:**
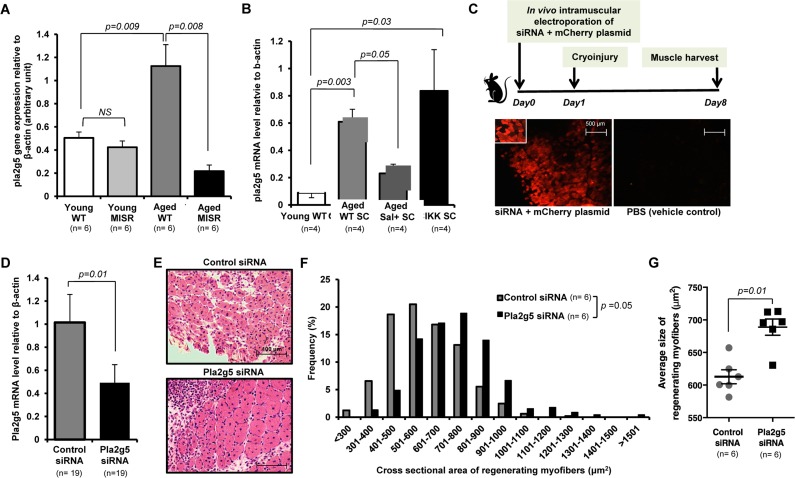
Inhibition of *pla2g5* expression improves muscle regeneration in aged mice (**A**, **B**) Expression of *pla2g5*, normalized to β-actin in (**A**) muscle tissues of young and aged WT and young and aged MISR mice (n=6 mice per experimental group), and (**B**) satellite cells isolated from young and aged WT, aged WT treated with sodium salicylate, and young SCIKK mice (n=4 mice per experimental group), determined by quantitative RT-PCR. Data presented as mean ± s.e.m.; p-values calculated by one-way ANOVA. (**C**) Experimental design. siRNA and mCherry plasmid were co-delivered to aged myofibers by *in vivo* electroporation. Muscles were damaged by cryoinjury 1 day after electroporation, and regenerating myofiber size was measured 7 days after cryoinjury. Electroporation efficiency in each sample was assessed by analysis of mCherry-expressing myofibers. Scale bars = 500 μm. (**D**) Efficiency of gene knockdown by *pla2g5* siRNA measured by qRT-PCR at muscle harvest and compared to levels of pla2g5 mRNA in muscles electroporated with control, scrambled siRNA (n=19 mice each group). Data represent mean ± s.e.m.; p-value calculated by Student's t test. (**E**) Representative H&E staining of muscle sections at day 7 after cryoinjury from aged mice receiving pla2g5 or control, scrambled siRNA. Scale bars = 100 μm. (**F**, **G**) Distribution and average of size of regenerating (centrally-nucleated) myofibers in aged mice receiving control, scrambled or pla2g5 siRNA (n=6 mice per experimental group). Contralateral TA muscles were used as controls with electroporation of scrambled siRNA. Data represented as histograms of fiber size (**E**) or as mean ± s.e.m. (**F**). P-values calculated by Kruskal-Wallis test for (**E**) and (**F**).

To identify factors that may mediate the NF-κB-regulated influence of the aged niche on muscle satellite cells, we performed a gene array study of whole skeletal muscle collected from young WT, aged WT, or aged MISR mice. Gene expression comparisons uncovered a group of genes that were expressed similarly in young WT and aged MISR mice, but differentially expressed in aged WT mice (Table S1). Many of these genes contained predicted NF-κB binding sites within their promoter regions [29] (Table S1). Among the candidates, PLA2G5 stood out as a secreted protein with functional similarity to snake venom myotoxins that can induce inflammation in neighboring cells [30,31]. Quantitative PCR analysis confirmed increased *pla2g5* expression in aged WT muscle and reduced expression in aged MISR muscle (Fig. 3A). Although present at substantially lower levels than in whole muscle tissue, *pla2g5* was also expressed in muscle satellite cells, with higher levels in aged WT and young SCIKK mice and lower levels in young WT and aged WT mice treated with salicylate (Fig. 3B). We therefore tested whether inhibition of *pla2g5* expression in muscle might be sufficient to restore muscle regeneration in aged mice.

Using *in vivo* electroporation [32], *pla2g5* siRNA was co-delivered with mCherry fluorescent protein-expressing plasmid into tibialis anterior (TA) muscles of aged mice (Fig. 3C). The contralateral muscles of the same mice were electroporated with a control siRNA (containing no significant sequence similarity to mouse, rat, or human gene sequences) (Fig. 3C). Electroporated muscles were cryoinjured 1 day after electroporation, and evaluated for regeneration after an additional 7 days. Electroporation efficiency in these studies ranged from ~30-70% (as determined by %mCherry^+^ fibers), and gene knockdown, measured at harvest, was 51.9% on average (S.D. ± 24.1, Fig. 3D). Thus, the level of reduction of *pla2g5* mRNA achieved in this system is comparable to the difference observed in skeletal muscle of young versus aged mice (see Fig. 3A). Muscle regeneration was markedly improved in *pla2g5* knockdown muscles, which showed improved histology (Fig. 3E) and an increased caliber of regenerating fibers (Fig. 3F,G). These data suggest that up-regulation of *pla2g5* may be one mechanism by which NF-κB activation in aged muscle fibers inhibits satellite cells locally and restrains muscle regeneration.

Overall, these *in vivo* experiments indicate in two complementary transgenic models (one gain-of-function and one loss-of-function) a cell-non-autonomous inhibitory effect of increased transcriptional activity of NF-κB in muscle fibers on satellite cell function, mediated at least in part by PLA2G5. These data additionally suggest that restraining NF-κB activity throughout life may protect against some age-acquired defects in satellite cell activity and muscle regeneration.

### Systemic sodium salicylate treatment improves myogenic function of aged satellite cells

To begin to evaluate the possible clinical utility of our observations, we next sought to determine whether acute, pharmacological inhibition of NF-κB signaling might also be effective for reversing age-related deficits in muscle repair. We therefore placed aged WT mice (24 months) on high-dose sodium salicylate or control diet for 6 weeks. Prior studies demonstrate that high-dose salicylate therapy is safe in mice and humans and effective in reducing NF-κB mediated inflammation [14,27,33]. In prior studies, systemic administration of sodium salicylate in mice decreased muscle atrophy caused by myofiber-restricted overexpression of IKKβ [14]. However, what role, if any, sodium salicylate might play in modulating muscle regeneration was not studied.

**Figure 4:**
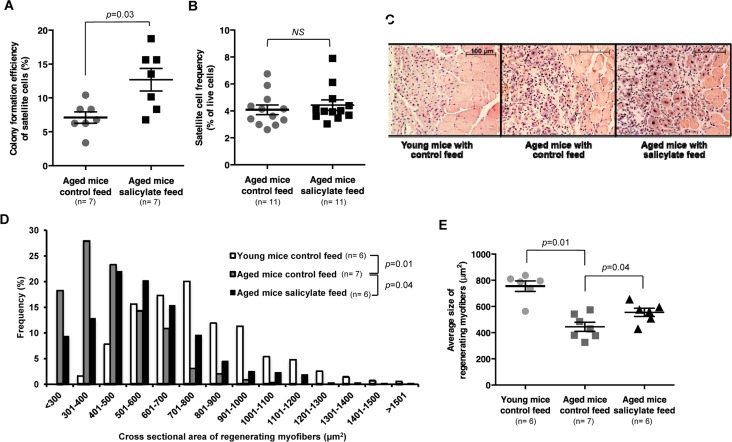
Sodium salicylate partially reverses aging-associated skeletal muscle inflammation and improves myogenic function of aged satellite cells (**A**) Myogenic colony forming efficiency of satellite cells from uninjured muscle of aged WT mice receiving control (n=7 mice) or salicylate feed (n=7 mice). (**B**) Satellite cell frequency (percent of live cells by flow cytometry) in aged mice receiving control feed versus salicylate feed (n=11 per group). Data presented as mean ± s.e.m.; p-value calculated by Student's t test for both (**A**) and (**B**). (**C**) Representative H&E staining of muscle sections taken 7 days after cryoinjury of young WT mice, or aged WT mice receiving control or salicylate feed for 6 weeks prior to injury. Salicylate treatment group continued on salicylate feed during recovery after injury. Scale bars = 100 μm. (**D**, **E**) Quantification of regenerating (centrally-nucleated) myofiber size at day 7 after cryoinjury in young WT mice or aged WT mice receiving control or salicylate feed for 6 weeks prior to injury (n=6 or 7 mice per experimental group). Salicylate treatment group continued on salicylate feed during recovery after injury. Data presented as a histogram of fiber size (**D**) or average fiber cross-sectional area (**E**). P-values were calculated by Kruskal- Wallis test and Step-down Bonferroni method for (**D**) and (**E**).

EMSAs performed on whole hind limb muscle demonstrated a 50% decrease in NF-κB activity in salicylate-treated aged mice (Fig. S8A,B), and satellite cells from these mice similarly displayed evidence of reduced NF-κB transcriptional response (Fig. S8C). Similar to results obtained in aged MISR mice, satellite cells from salicylate-treated aged WT mice showed improved myogenic colony forming ability (Fig. 4A), but no change in satellite cell frequency (Fig. 4B, Fig. S9). Aged mice receiving sodium salicylate also showed improved muscle regeneration at early time points after cryoinjury (day 7 post-injury, Fig. 4C-E), although this difference was lessened at later time points (day 14 post-injury; Fig. S10). In contrast, young mice and aged MISR mice given sodium salicylate did not exhibit noticeable differences in muscle regeneration after cryoinjury or in the in vitro myogenic activity of satellite cells (Fig. S11, S12, Fig. 1F,G), as expected given their low basal levels of NF-κB activity (Fig. S2, S6).

## DISCUSSION

Taken together, data presented here suggest that chronic inflammatory signaling in muscle fibers, mediated by NF-κB, retards the myogenic potential of satellite cells in aged muscle. Although NF-κB target genes are activated in satellite cells of aged mice, and in mice harboring a satellite cell-specific IKKβca transgene, these satellite cells exhibited no apparent cell-intrinsic defects in frequency or function. Defective satellite cell function in the context of induced NF-κB activity was revealed only when these cells were exposed to mature muscle fibers or myotubes harboring heightened NF-κB signaling (either in aged mice or when SC-IKK nuclei were incorporated into regenerating myotubes/myofibers during muscle differentiation and repair). Importantly, these results should not be taken as evidence that satellite cells are completely unaffected by cell-intrinsic elevation of NF-kB signaling. Indeed, activation of NF-kB in young satellite cells clearly alters their transcriptional program (Fig. 1B) and perturbs their performance in differentiation cultures, which could reflect either cell autonomous or non-autonomous effects (see Fig. S5). Nonetheless, our results are highly consistent with a critical role for cell-extrinsic regulation of satellite cell function in these contexts, a notion that is further supported by the observation that aged MISR mice, which maintain dominant inhibition of NF-κB function in muscle fibers alone, are partially protected from age-dependent loss of satellite cell function.

Our data further suggest that NF-κB may exert its effects via PLA2G5, a secreted phospholipase whose expression is increased in aged muscle fibers. PLA2G5 is known to act on lipoprotein and phosphomembrane substrates and can release key lipid mediators including free fatty acids, lysophospholipids and arachidonic acid, a precursor to eicosanoid signaling [34]. Thus, increased PLA2G5 in muscle may act directly on satellite cell membranes, or indirectly on acellular lipids or membranes of other cell types residing in the satellite cell niche, to liberate lipid signaling molecules that inhibit muscle regeneration. While determination of the precise lipid mediators involved remains a subject for future studies, it is intriguing to note that elevated levels of prostaglandin E2 (PGE2), a classic eicosanoid produced by the sequential action of cyclooxygenase and terminal prostaglandin E synthase on arachidonic acid, have been reported to contribute to defective differentiation of human myoblasts from myotonic dystrophy type 1 (DM1) patients with large CTG repeat expansions [35]. Interestingly, levels of PGE2 are also elevated in muscle extracts from aged mice (Fig. S13), consistent with an enhanced production of the PGE2 precursor arachidonic acid as a result of increased expression by aged muscle fibers of PLA2G5. However, as the specific effects of PGE2 on satellite cells and muscle regeneration were not evaluated here, the impact on muscle aging of this particular effector, and of the multitude of other lipid mediators generated by PLA2G5, remain important topics for future investigation.

Our results are consistent with prior studies implicating NF-κB induction in diminished muscle regeneration in cachectic [5] and dystrophic [17] mice, and in response to the pro-inflammatory cytokine TWEAK [36]. Of particular note, a prior report showed that transplanted satellite cells contribute less robustly to muscle repair when these cells are introduced into the muscle of tumor-bearing mice, which have increased NF-κB activity in myofibers [5], although this study did not specifically distinguish cell-intrinsic from extrinsic contributions of NF-κB signaling in this context. Another study evaluated NF-κB activity in the dystrophic muscles of *mdx* mice, and found that conditional deletion of IKKβ in activated macrophages or muscle fibers impeded regeneration in this system [37]. Our study similarly reveals a non-cell-autonomous regulation of satellite cell regenerative function by NF-κB driven gene expression in myofibers in aged muscle, and identifies PLA2G5 as a potential novel mediator of this effect. Whether PLA2G5 is similarly induced in cachectic and dystrophic muscle, as it is in aging muscle, will be an interesting area for future research. It will also be important to further interrogate the molecular circuitry that drives elevated NF-κB activity in aged muscle fibers, possibly through the generation of inducible alleles that allow perturbation of various NF-κB signaling components in aged myofibers, and to determine whether other NF-κB driven mechanisms, such as elevated TNFα or IL-6 production, implicated in the deregulation of satellite cell function in dystrophic and cachectic muscle, also contribute to satellite cell dysfunction in aged muscle [38]. This is particularly relevant, as increased circulating levels of TNFα and IL-6 have been reported in aged individuals [39,40].

The role of anti-inflammatory therapy for the treatment of skeletal muscle dysfunction in elderly patients is a subject of ongoing investigation [41,42]. It has been shown that, following 12 weeks of exercise, NSAID therapy modestly increased muscle strength without hypertrophy in patients with osteoarthritis of the knee [43]. Trappe et al. have further suggested that NSAID treatment enhances muscle mass and strength during resistance training in older individuals, potentially by decreasing PGE2 levels in skeletal muscle [41,44,45]. Finally, our observation that high-dose sodium salicylate improves regeneration of aged muscle suggests that, similar to Losartan, the only other FDA-approved drug that has shown promise at a basic science level as a therapeutic agent [46], salsalate should similarly be considered as a potential pharmacologic therapy to boost recovery after muscle damage in elderly individuals.

## MATERIALS AND METHODS

### Animals

Young (2–3 month) and aged (24 month) C57BL/6 mice were obtained from pathogen-free breeding colonies at Jackson Laboratories and Charles River Labs. MISR (a kind gift from S. Shoelson [14], and Pax7Cre-ER mice (a kind gift from C. Keller [25] were bred in house; loxP-STOP-loxP-IKKβ (C57BL/6-Gt(ROSA)26Sor^tm1(Ikbkb)Rsky^/J) mice were purchased from Jackson Labs. Fidelity of the Pax7-CreER line for satellite cell-specific gene expression has been validated previously [25]. Tamoxifen (0.1 mg /g body weight, Sigma Aldrich) or corn oil vehicle (10 μl /g body weight) was injected for 5 consecutive days. For sodium salicylate treatment, a feed containing 3 grams of sodium salicylate (Sigma Aldrich) per kg was created by Research Diets, and purchased along with control feed containing the same nutritional content but lacking salicylate. Mice were provided sodium salicylate or control feed ad libitum for 6 weeks prior to experimen-tation, including two weeks prior to tamoxifen treatment. For injury experiments on salicylate-treated mice, animals were continued on salicylate feed during recovery after injury (7 days or 14 days). Animals were housed at the Biomedical Research Institute at Harvard University and the animal facility at the Joslin Diabetes Center. Animal procedures conducted in this study were reviewed and approved by Institutional Animal Care and Use Committees (IACUC) at Harvard's Faculty of Arts and Sciences and at the Joslin Diabetes Center.

### Satellite cell isolation

Single myofibers and myofiber-associated cells were prepared from two-step collagenase/dispase digestion of intact limb muscles (EDL, gastrocnemius, quadriceps, soleus, TA, and triceps brachii), as previously described [4,21,47,48]. All myofiber-associated cells were incubated in Hank's Buffered Salt Solution (Gibco) containing 2% donor bovine calf serum on ice for 20 min with the following antibodies: anti-mouse CD45 (1:200, clone 30-F11, Biolegend Cat #103106 for PE conjugate, or Biolegend Cat#103115 for APC/Cy7 conjugate); anti-mouse/human CD11b (1:200, clone M1/70, Biolegend Cat# 101208 for PE conjugate, or Biolegend Cat#101226 for APC/Cy7 conjugate); anti-mouse Ter119 (1:200, Biolegend Cat#116208 for PE conjugate, or Biolegend Cat#116223 for APC/Cy7 conjugate); APC conjugate anti-Ly-6A/E (Sca-1) (1:200, clone D7, Biolegend #108112); anti-mouse CD29 (β1-integrin) (1:200, clone HMB1-1, BD Pharmingen Cat# 102202; or 1:100, Biolegend Cat#102208 for PE conjugate); FITC conjugate anti-armenian hamster IgG (1:100, eBioscience Cat#11-4111-85); biotinylated anti-mouse CD184 (CXCR4) (1:100, BD Pharmingen Cat#551968); and PE/Cy7 conjugate anti-streptavidin (1:100, eBioscience Cat#25-4317-82). Muscle satellite cells, identified as CD45^−^ Sca-1^−^ CD11b^−^ Ter119^−^CXCR4^+^ β1-Integrin^+^ cell population as in previous studies [4,21,47,48]. Cells were sorted by Fluorescence Activated Cell Sorting (FACS) using Aria II (BD Biosciences). Live cells were identified as calcein blue positive (1:1000, Invitrogen) and propidium iodide negative (PI, 1mg/mL, Sigma Aldrich). Satellite cells were double-sorted to maximize the purity of sorted cells [4,21,47,48]. Previous studies have shown that >90% of CD45^−^ Sca-1^−^ Mac-1^−^ CXCR4^+^ β1-integrin^+^ cells express Pax7, the canonical satellite cell marker, and that this marker profile identifies >98% of Pax7^+^ satellite cells [4,21,47,48,49]. Flow cytometry and cell sorting were performed at the Joslin Diabetes Center or HSCRB Flow Cytometry Cores. Sorting and analysis were carefully optimized for antibody titration and to achieve maximal cell purity and viability [4,21,47,48].

### Myogenic colony-forming assays

A single CD45^−^ Sca-1^−^ Mac-1^−^ CXCR4^+^ β1-integrin^+^ satellite cell was seeded in each well of a 96-well plate coated with PBS containing collagen (1mg/ml, Sigma) and laminin (10mg/ml, Invitrogen) for at least an hour at 37°C. Cells were cultured in F10 medium containing 20% horse serum, 1% glutamax, and 1% Penstrep, and supplemented daily with 5ng/mL bFGF (Sigma Aldrich), as previously described [4,21,47]. Colony formation efficiency was analyzed on day 5 of culture and reported as the percent of seeded wells that contained cell colonies. The number of cells per colony was also quantified in some experiments. All cell colonies that arise in these assays are myogenic. No fibroblast or adipocyte colonies were detected or scored [4,21,47].

### *In vivo* electroporation

TA muscles subjected to in vivo electroporation were pre-conditioned by injection of 10 ul of 2 mg/ml hyaluronidase solution 1 hour prior to electroporation. A mixture of 50 pmol of siRNA (Life Technologies) and 30 μg of mCherry-expressing plasmid (Addgene) in 10 ul of PBS was injected into the pre-conditioned TA muscles 10 minutes before electroporation. Following injection, electric pulses were delivered using an electric pulse generator (Electro Square porator ECM 830; BTX), by a pair of electrodes at the site of injection. Ten pulses were delivered, each with 20 ms duration at 1Hz. This procedure was repeated with negative control siRNA (Life Technologies) on the contralateral muscle.

### Quantitative RT-PCR

For mRNA extraction, CD45^−^ Sca-1^−^ Mac-1^−^ CXCR4^+^ β1-integrin^+^ satellite cells harvested from mouse skeletal muscle were double-sorted for purity, and deposited in Trizol (Invitrogen). For whole muscle mRNA, muscle was homogenized in Trizol using a Gentle MACS Dissociator (Miltenyi Biotech). cDNA was prepared from mRNA using Superscript III Reverse Trans-criptase Supermix Kit (Invitrogen). Real-time quantitative PCR reactions were carried out in an ABI 7900 machine, using SYBR Green PCR mix (Applied Biosystems). β-actin was used as a housekeeping gene, and gene expression levels were normalized to β-actin expression. Primers sequences are provided in Supplementary Table 2.

### Electrophoretic mobility shift assay (EMSA)

All nuclear extraction procedures were performed on ice with ice-cold reagents. Nuclear protein was extracted using the Nuclear Extraction Kit (Panomics). Protein concentration of nuclear extract was measured using the DC Protein Assay (BioRad). Binding tests for transcription factors were performed as described by the manufacturer (Affymetrix). 10 μl of binding reaction mixtures containing 1 μg of poly(dI–dC) and 10 ng biotin-labeled transcription factor probe in binding buffer were incubated with 5 μg cell nuclear extracts at 15°C for 30 min, followed by fractionation on native 5% TBE polyacrylamide gels, transfer to a nylon membrane, and detection by streptavidin HRP solution. Results were visualized by autoradiography and quantified using Image J densitometry.

### Cryoinjury of muscle and quantification of cross sectional area of regenerating myofibers

For cryoinjury, mice were anesthetized and dry ice was applied directly to the exposed TA muscle for 5 seconds. The skin incision was closed with suture immediately after injury. This procedure generates a reproducible injury in the muscle with a discrete border between uninjured and injured muscle, and this border remains clear and distinct during regeneration of the injured tissue [1,3,4,50]. Injured muscles were allowed to recover for 7 or 14 days before harvest. For quantification of regenerating myofiber size after cryoinjury, a series of pictures were taken spanning the entire regenerating area in cross section, and the sizes of 15 regenerating myofibers (identified by their centrally located nuclei) were measured in each image, which collectively resulted in total of ~150 myofiber sizes measured for each animal using Axiovert software.

### RNA transcriptome analysis

Total RNA was extracted from muscle tissue using Trizol (Invitrogen). RNA quality was assessed with a 2100 Bioanalyzer (Agilent Technologies), and samples with RNA Integrity Number (RIN) higher than 8.5 were used. The total RNA samples were pre-processed using the GeneChip WT PLUS Reagent Kit (Affymetrix) following the manufacturer's protocol for hybridization to GeneChip Mouse Gene 2.0 ST Arrays (Affymetrix) by the Boston Children's Hospital Intellectual and Developmental Disabilities Research Center (IDDRC) Molecular Genetics Core Facility. The dataset was normalized by the robust multi-array average (RMA) algorithm [51] and analyzed with the R software (www.r-project.org). The dataset has been deposited at NCBI's Gene Expression Omnibus (GEO) and are accessible through GEO Series accession number GSE72179. Genes which were expressed similarly in young WT and aged MISR mice, but differentially expressed in aged WT mice, were chosen based on fold-change as candidates. Among candidates, genes found in the literature to have potential to mediate NF-κB signaling during inflammation were assayed by qRT-PCR for validation (data not shown). Candidate genes validated for their expression pattern in muscle tissues of young and old WT and MISR mice are shown in Supplementry Table 1. In addition, candidate genes were also evaluated for NF-κB binding prediction index obtained by transcription factor binding site analysis by MatInspector computational software (www.genomatix.de). For NF-κB binding prediction index, “matrix similarity” between the promoter sequence of each candidate gene and the position weight matrices (the complete nucleotide distribution for each single position) of the DNA-binding motif of NF-κB was calculated by MatInspector as previously described [29]. A perfect match to the matrix gets a score of 1.00 (each sequence position corresponds to the highest conserved nucleotide at that position in the matrix) [29].

### Histology

Harvested muscles were fixed in 4% paraformaldehyde, washed in PBS and stored in 70% ethanol for paraffin embedding, or frozen for cryo-sectioning (10μm sections). Hematoxylin and eosin (H&E) staining was used to visualize regenerating myofibers in injured muscles.

### Microscopy

Bright field images were acquired using Carl Zeiss Axiovert 40C and Carl Zeiss Observer D1 (Carl Zeiss Inc.). Fluorescence images were acquired using Carl Zeiss Axio Imager M1 (Carl Zeiss Inc.).

### Prostaglandin E2 ELISA

Muscle extract samples for ELISA were obtained by homogenizing quadriceps muscles of young and aged mice in homogenization buffer (0.1 M phosphate, pH 7.4, containing 1mM EDTA and 1uM indomethacin). Prostaglandin E2 ELISA was performed according the protocol provided by the manufacturer (Abcam, cat# ab133021).

### Statistical analyses

Data are presented as mean ± standard error of mean (s.e.m.). For all graphic data, *n* indicates the number of biological replicates. The number of animals used per group was determined based on accumulated empirical data in the laboratory and anticipated robustness of the data points [4,21,47]. The number of animals for each experiment was appropriate to detect the differences, if present, in the experimental outcomes. For salicylate feed treatment and *pla2g5* knockdown experiments, animals were randomly allocated for different treatment conditions. Animals diagnosed with severe health concerns (tumors, malocclusion, etc.) by veterinary staff were excluded from analysis; this exclusion criterion was pre-determined. Data were tested for normal distribution, and the observed variation was similar between groups. Statistical comparisons for normally distributed data were performed using appropriate tests, as indicated below. Results comparing two different groups were assessed for statistical significance using Student's *t* test (Microsoft Excel; GraphPad Prism, GraphPad Software Inc.) assuming two-tailed distribution (Figure 1H,I; Figure 2E; Figure 3D,G; Figure 4A,B) and results comparing more than two groups were assessed by one-way ANOVA with Turkey's multiple comparison test (GraphPad Prism, GraphPad Software Inc.; Figure 1B,F,G; Figure 2D; and Figure 3A,B). For statistical analyses of distribution and average of regenerating myofiber sizes in injured muscles, p-values were calculated by Kruskal-Wallis test and adjusted, if necessary, by Stepdown-Bonferroni method (Figure 1C,D; Figure 2A,B; Figure 3F,G; and Figure 4D,E). Investigators were blinded to experimental group assignment for outcome assessment. Statistical significance was accepted at p < 0.05.

## SUPPLEMENTARY MATERIAL FIGURES AND TABLES


