# Supplementary material for: Age-associated NF-κB signaling in myofibers alters the satellite cell niche and re-strains muscle stem cell function
Source: Aging (Albany NY). 2016 Nov 14;8(11):2871–84. doi: 10.18632/aging.101098 (PMC5191876; doi:10.18632/aging.101098)
Supplement: Supplementary file 1 [file aging-08-2871-s001.pdf]

## SUPPLEMENTARY MATERIAL

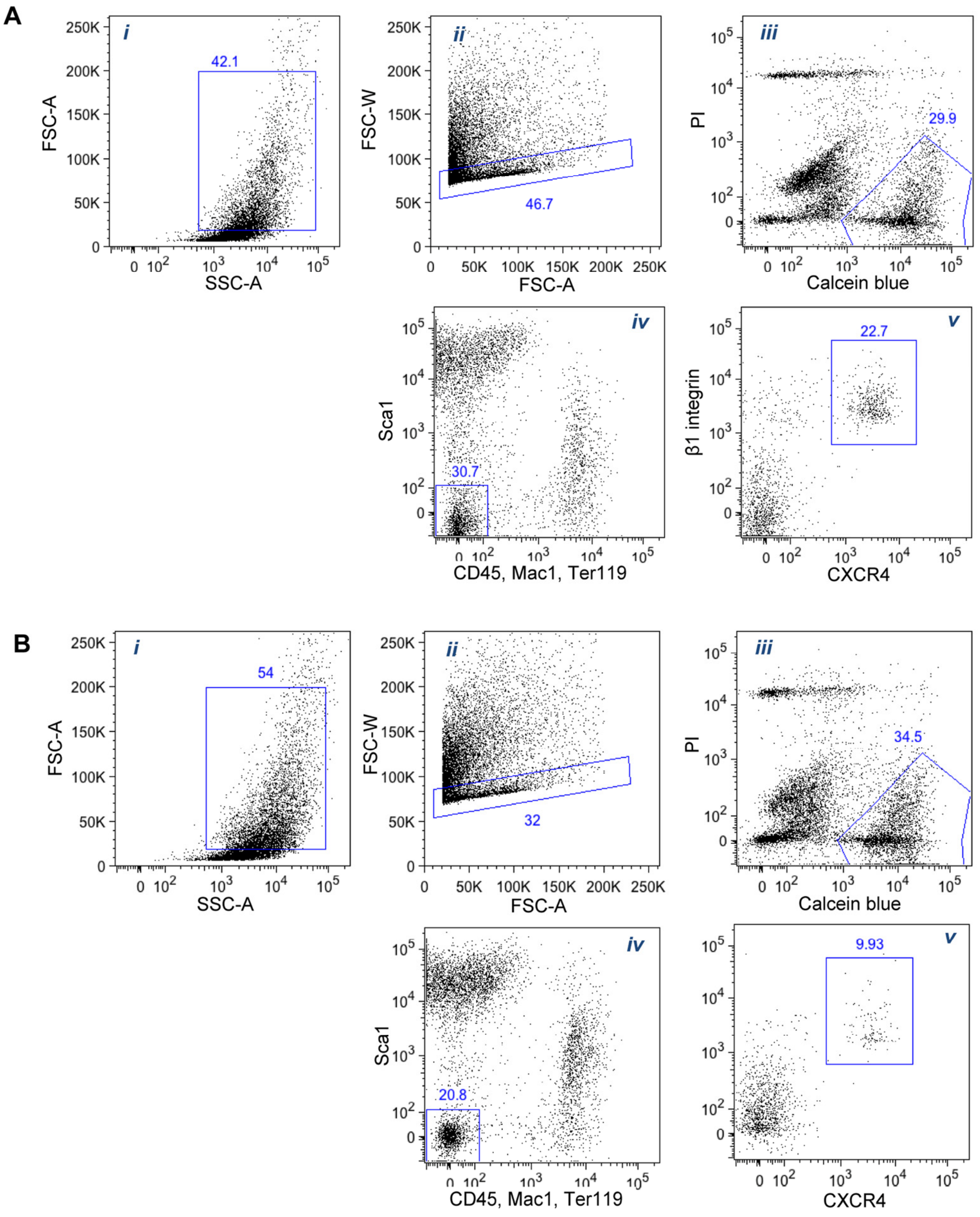


**Figure S1. Cell sorting strategy and reduced frequency of satellite cells among myofiber-associated mononuclear cells in young versus aged mice.** Myofiber-associated cell sorting by flow cytometry was performed as previously described [4, 21, 42, 43]. Representative flow plots depict the sequential gating strategy *(i, ii, iii, iv, v)* utilized and in young (A) and aged (B) mice. Numbers represent percentage of previously gated population. Young mice ranged in age from 2-3 months. Aged mice were 24 months old. Old mice demonstrate decreased frequency of CD45-Sca1-Mac1-CXCR4+β1integrin+ satellite cells.

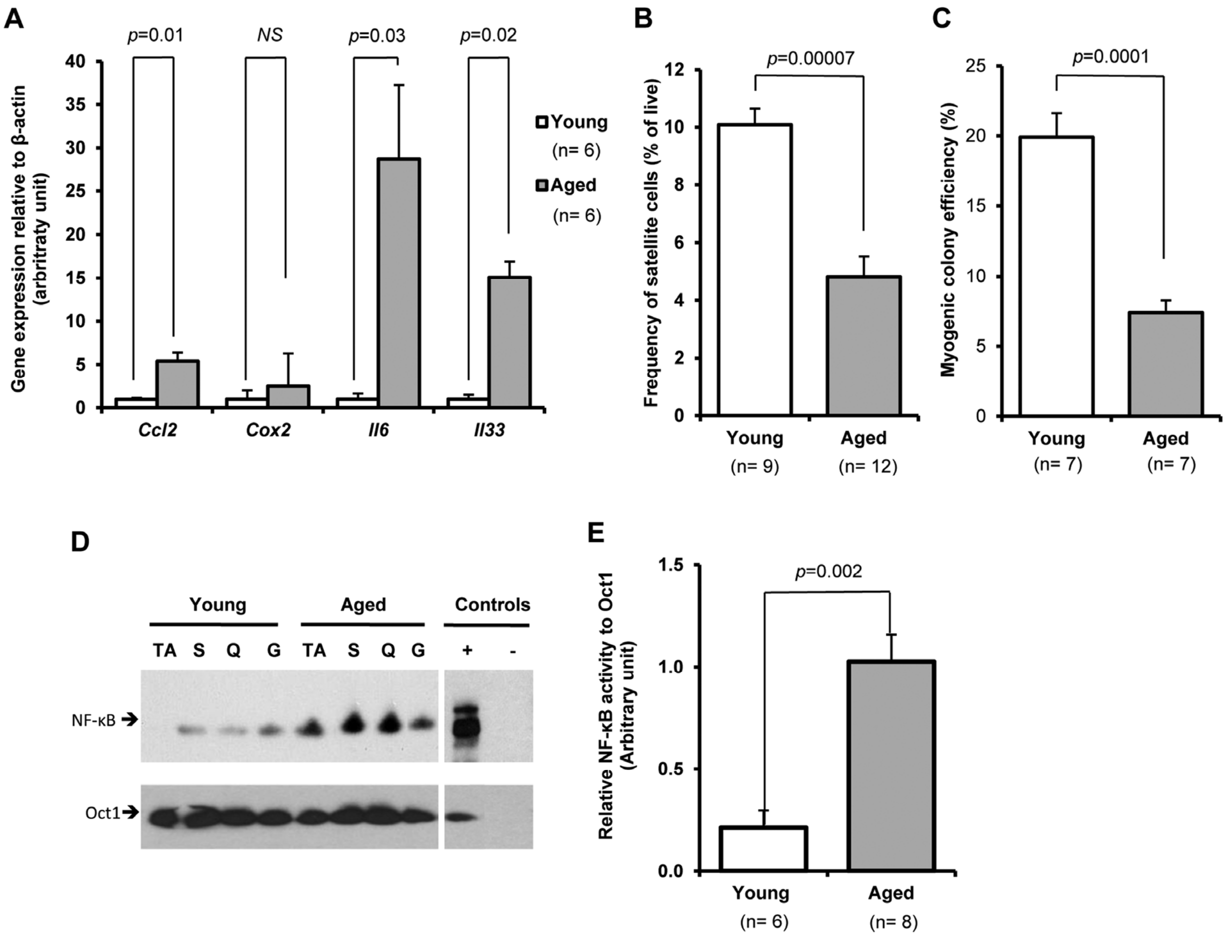


**Figure S2. Satellite cell frequency and function is reduced with aging.** (**A**) Flow cytometric analysis demonstrates an ~2-fold decrease in the frequency of satellite cells in aged mice (n=12) as compared to young mice (n=9). (**B**) Double-sorted satellite cells from aged mice (n=7) exhibit a 3-fold decrease in myogenic colony-forming efficiency as compared to cells isolated from young mice (n=7). (**C**,**D**) EMSA reveals increased NF-kB activity in hind limb skeletal muscle of aged (n=8) as compared to young mice (n=6). Data are summarized for analysis of fast and slow-twich hindlimb muscles in (**C**) and representative EMSA of slow-twitch (soleus, S) and fast twitch (tibialis anterior, TA; quadriceps, Q; gastrocnemius, G) hind-limb skeletal muscles is shown in (**D**). Oct1 was used as a loading control. (**E**) Quantitative RT-PCR analysis of inflammatory genes related to the NF-κB pathway in satellite cells from young or aged mice (n=4-6 samples per gene). *Ccl-2*, *Cox-2*, and *Il-6* are transcriptionally upregulated by NF-κB, whereas *Il-33* mediates increased NF-kB activation. Young mice ranged in age from 2-3 months. Aged mice were 24 months old. All data plotted as mean ± s.e.m. Student's *t* test was used for statistical analysis. *NS*: not statistically significant.

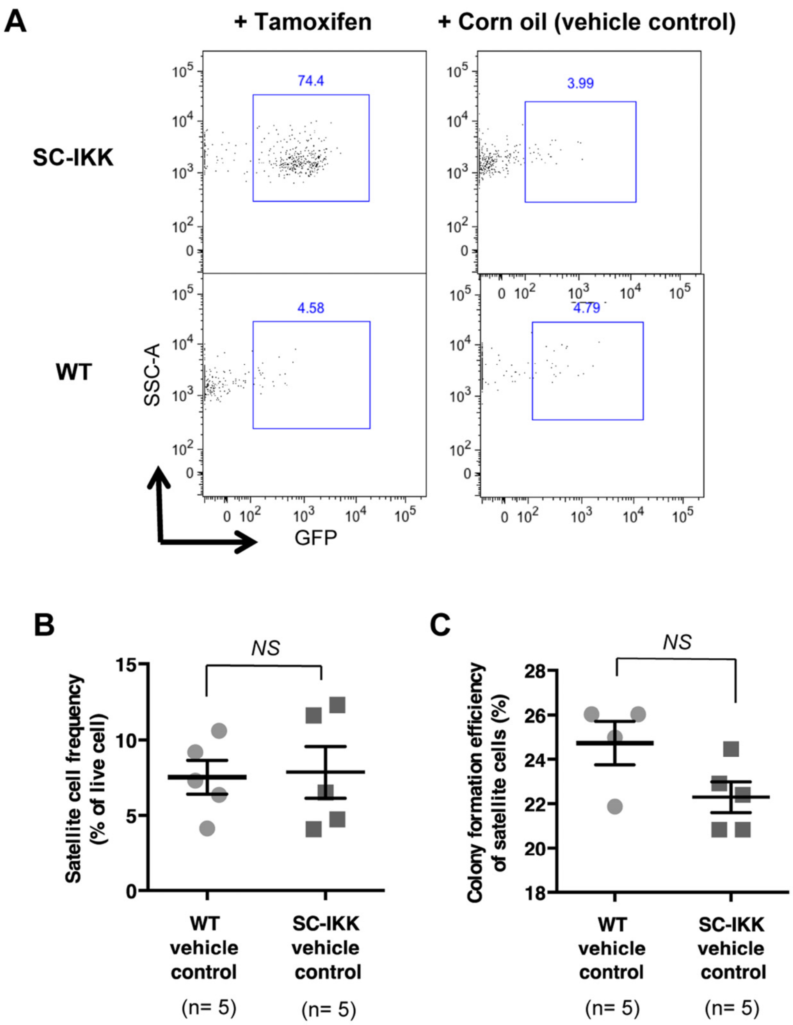


**Figure S3. Satellite cell-specific activation of NF-κB activity.** (**A**) Satellite cells from tamoxifen treated SC-IKK mice show transgene-induced GFP expression, which is not seen in vehicle treated SC-IKK controls. GFP+ cells also were not detected in other cellular compartments (CD45+ immune cells and Sca-1+ fibro/adipogenic lineage cells) in muscle (data not shown). Satellite cell frequency (**B**) and myogenic colony formation efficiency (**C**) are unperturbed in WT or SC-IKK mice injected with vehicle (corn oil) only (n=5). For these experiments, all mice received tamoxifen (TAM) or vehicle at 8-9 weeks of age. All data plotted as mean ± s.e.m. Student's *t* test was used for statistical analysis. *NS*: not statistically significant.

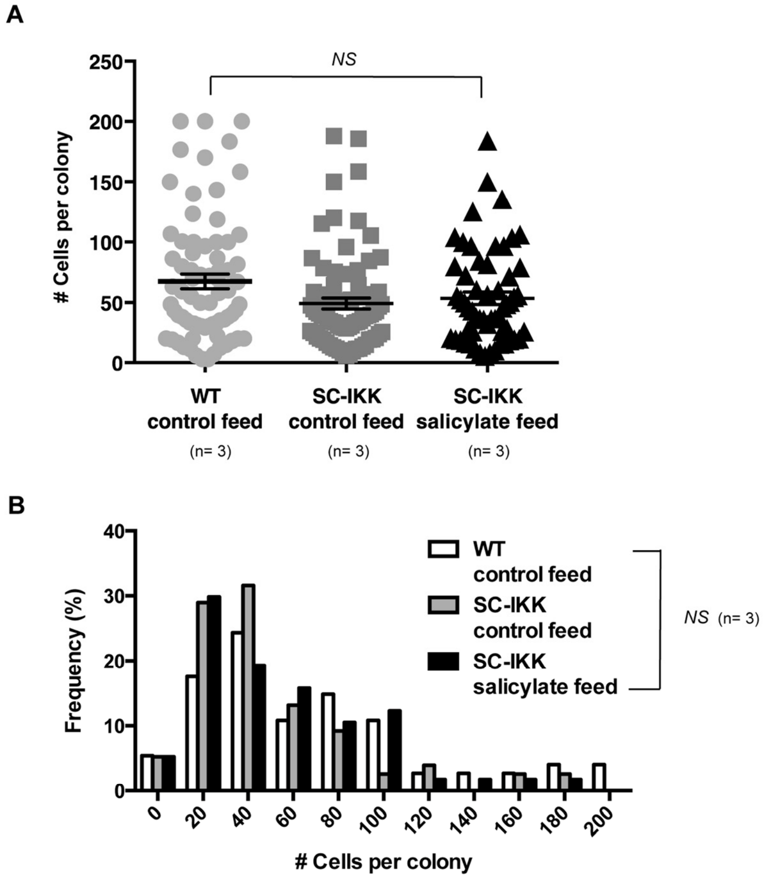


**Figure S4. Satellite cells isolated from SC-IKK mice show no difference in their intrinsic ability to form myogenic colonies in single-cell culture.** (**A**) Number of cells in each myogenic colony formed in single cell culture of satellite cells isolated from WT mice or SC-IKK mice with or without sodium salicylate treatment. Data plotted as mean ± s.e.m. Student's *t* test was used for statistical analysis. (**B**) Frequency of myogenic colonies of different sizes formed in single cell culture of satellite cells isolated from WT mice or SC-IKK mice with or without sodium salicylate treatment. For these experiments, all mice received tamoxifen or vehicle at 8-9 weeks of age. *NS*: not statistically significant.

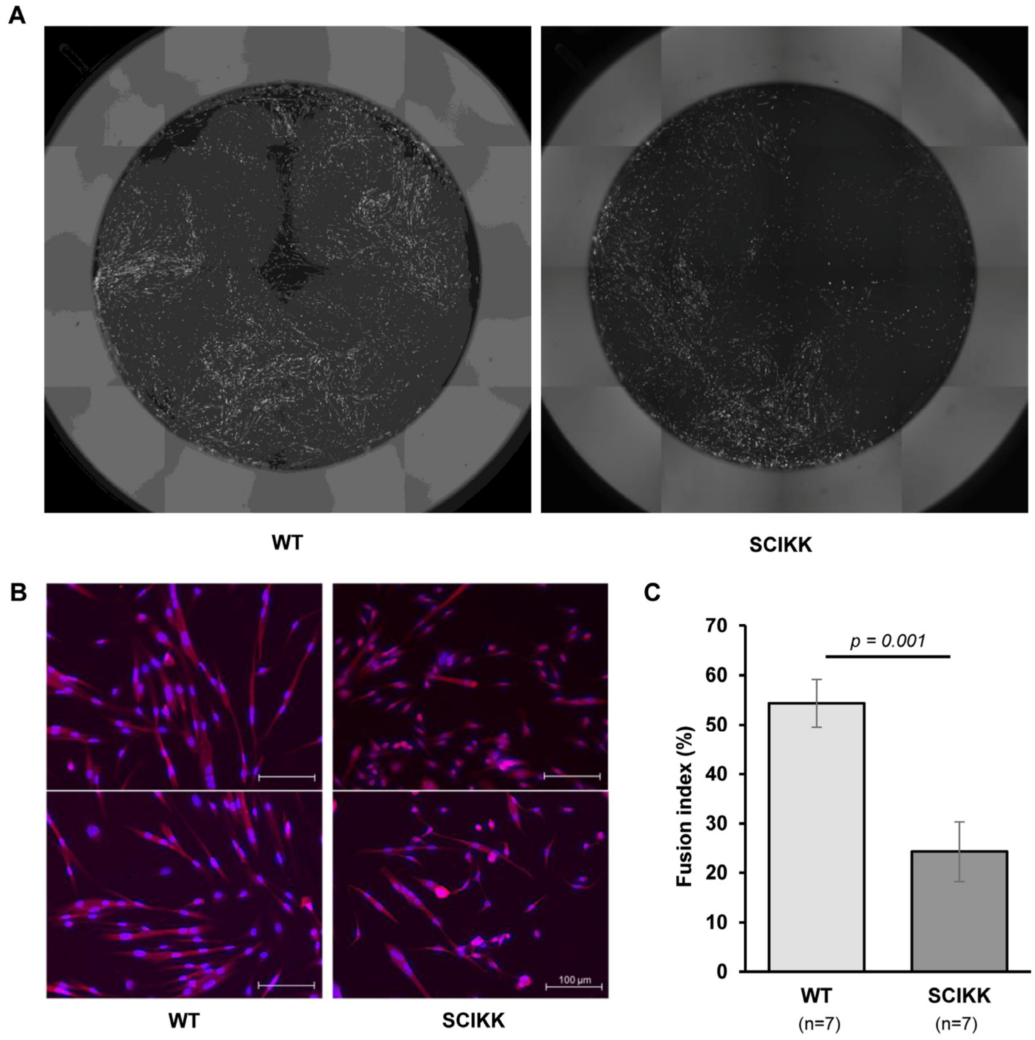


**Figure S5. In vitro differentiation of satellite cells from SCIKK mice produces cultures with fewer nuclei, smaller myotubes and a reduced fusion index.** (**A**) Representative images of muscle cell cultures initiated by satellite cells sorted from SC-IKK (right) or WT (left) mice. FACS-sorted satellite cells were seeded in 96-well plates at 1000 cells/well and cultured for 8 days. Resulting cultures were stained for nuclei and myosin heavy chain for visualization of differentiated muscle cells. Merged image of nuclei staining and myosin heavy chain staining was obtained for each well (shown above). Cultures contained both mononuclear cells and multinucelated myotubes. Cultures of SC-IKK cells contained fewer nuclei as compared to cultures of WT cells, as quantified at day 8 of culture. Image acquisition and cell number quantification (presented in Fig. 1H) of each well was performed by Celigo. (**B**) Representative images of cultures of *in vitro* differentiated satellite cells from SCIKK or WT mice 1000 cells were sorted into each well of a 96-well plate, proliferated for 5 days, and induced to differentiate by switching to low mitogen media (2% horse serum in F10 media). Cultures were stained after 2 days in differentiation media (Red: Myosin heavy chain; Blue: DAPI; Representative of 3 experiments). (**C**) Fusion index of WT satellite cell culture and SCIKK satellite cell culture indicates reduced differentiation capacity of SCIKK satellite cells. Fusion index was calculated as percentage of cell nuclei contained in multinucleated myotubes out of total number of nuclei. Thus, early differentiating cells in SCIKK cultures would produce NF-kB activated myoblasts and myotubes that could exert a cell-non-autonomous negative influence on the remaining, undifferentiated SCIKK satellite cells. These data may be consistent therefore with either cell autonomous or non-autonomous effects.

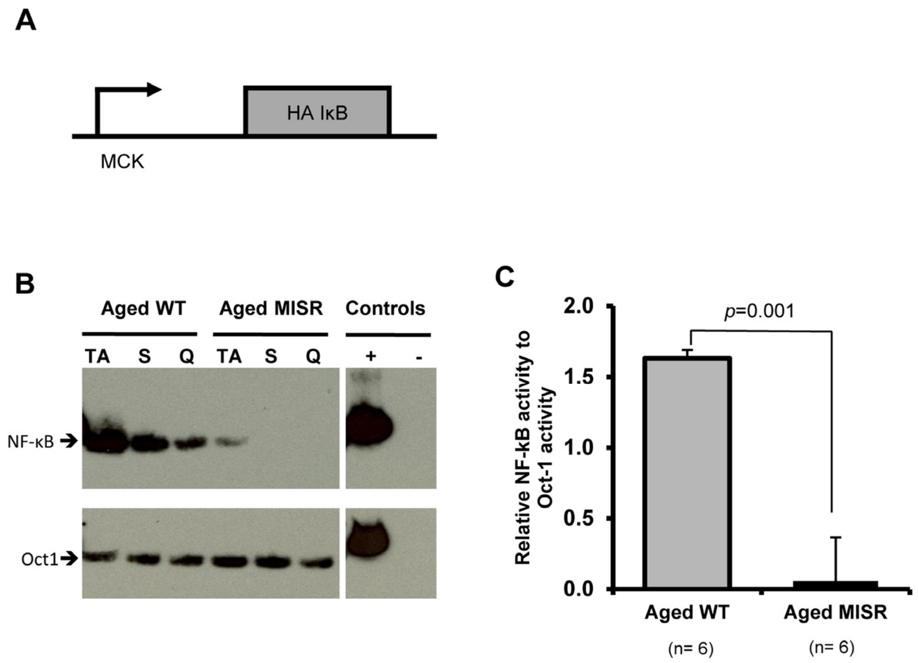


**Figure S6. MISR mice exhibit nearly complete inhibition of NF-κB activity even in old age.** (**A**) MISR mice carry a constitutively active, hyaluronic acid (HA) tagged, IκB gene under control of the muscle fiber-specific MCK promoter, and thus exhibit diminished NF-κB activity in a myofiber-specific fashion. (B) Representative EMSA, quantified in (**C**) for n=6 mice per group, demonstrating minimal NF-κB activity in the hind limb muscle of aged MISR mice (24 month). Oct1 was used as a loading control. All data plotted as mean ± s.e.m. Student's *t* test was used for statistical analysis.

**Table S1.** Genes that were differentially regulated in skeletal muscle fibers between the group of young WT and aged MISR mice as compared to aged WT mice were identified by Affymetrix gene chip array and validated by qRT-PCR. **Table 1a** identifies genes that exhibit higher expression in the myofibers of aged WT mice as compared to young WT or aged MISR mice. Conversely, **Table 1b** lists genes that demonstrate higher skeletal muscle fiber expression in young WT and aged MISR mice as compared to aged WT mice. NF-κB binding prediction index for each candidate gene was obtained by transcription factor binding site analysis by MatInspector computational software (www.genomatix.de).

| 1a. Genes up-regulated in aged WT, down-regulated in young WT, and down-regulated in aged MISR | NF-κB binding prediction index |
|---|---|
| ***Tmem100*** | **0.86** |
| ***Grem2*** | N/A |
| ***Pgpep1l*** | **0.96** |
| ***Fgfbp1*** | N/A |
| ***Mab21l1*** | N/A |
| ***Kcnf1*** | N/A |
| ***Scn3b*** | **0.93** |
| ***Aebp1*** | N/A |
| ***Kcnab1*** | **0.91** |
| ***Mir1a-1*** | **0.89** |
| ***Pla2g5*** | **0.94** |
| ***Dkk3*** | **0.84** |
| ***Pla2g7*** | **0.81** |
| ***Cdkn2c*** | N/A |
| ***Cadm1*** | **0.87** |
| ***Ostn*** | N/A |

| 1b. Genes down-regulated in aged WT, up-regulated in young WT, and up-regulated in aged MISR | NF-κB binding prediction index |
|---|---|
| ***Ccdc80*** | **0.92** |
| ***Agbl1*** | N/A |
| ***Nmrk2*** | **0.99** |
| ***Sacs*** | **0.86** |
| ***Plekho1*** | N/A |
| ***Retnla*** | **0.83** |
| ***Terc*** | **0.98** |
| ***Nr4a3*** | **0.86** |

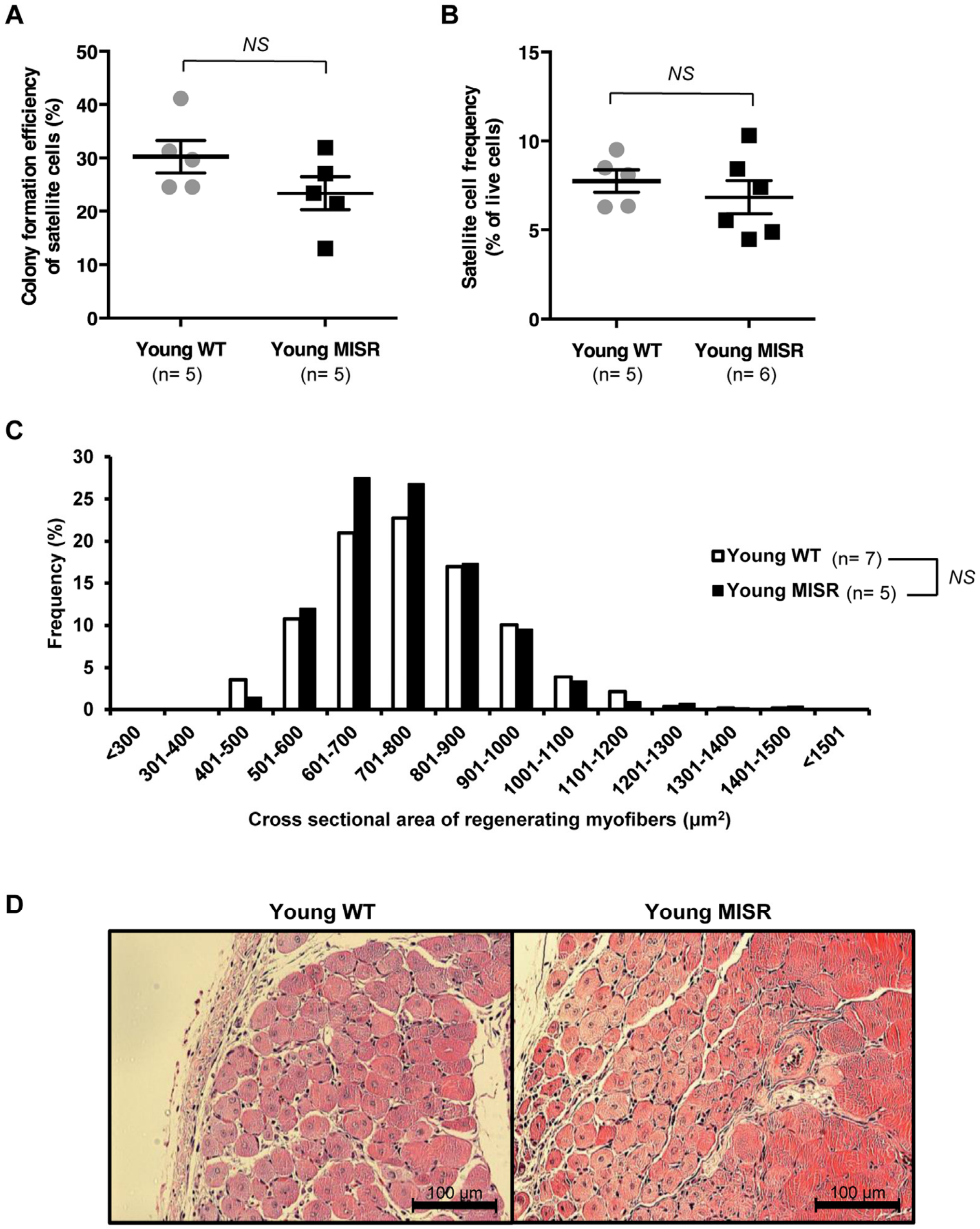


**Figure S7. Satellite cell function and muscle regeneration in young MISR mice (2-3 month).** (**A**, **B**) Young WT (n=5) and young MISR mice (n=5) show no difference in myogenic colony formation efficiency (*p*=0.15) or frequency of satellite cells (*p*=0.45). Data presented as mean ± s.e.m. (**C**) Skeletal muscle regeneration at 7 days after dry ice injury was similar between young WT (n=7) and young MISR (n=5) (*p*=0.65). (**D**) Representative cross sectional view of injured TA muscle of young WT and young MISR (H&E staining). NS: not statistically significant. Student's *t* test (**A**, **B**) and Kruskal-Wallis test (**C**) was used for statistical analysis.

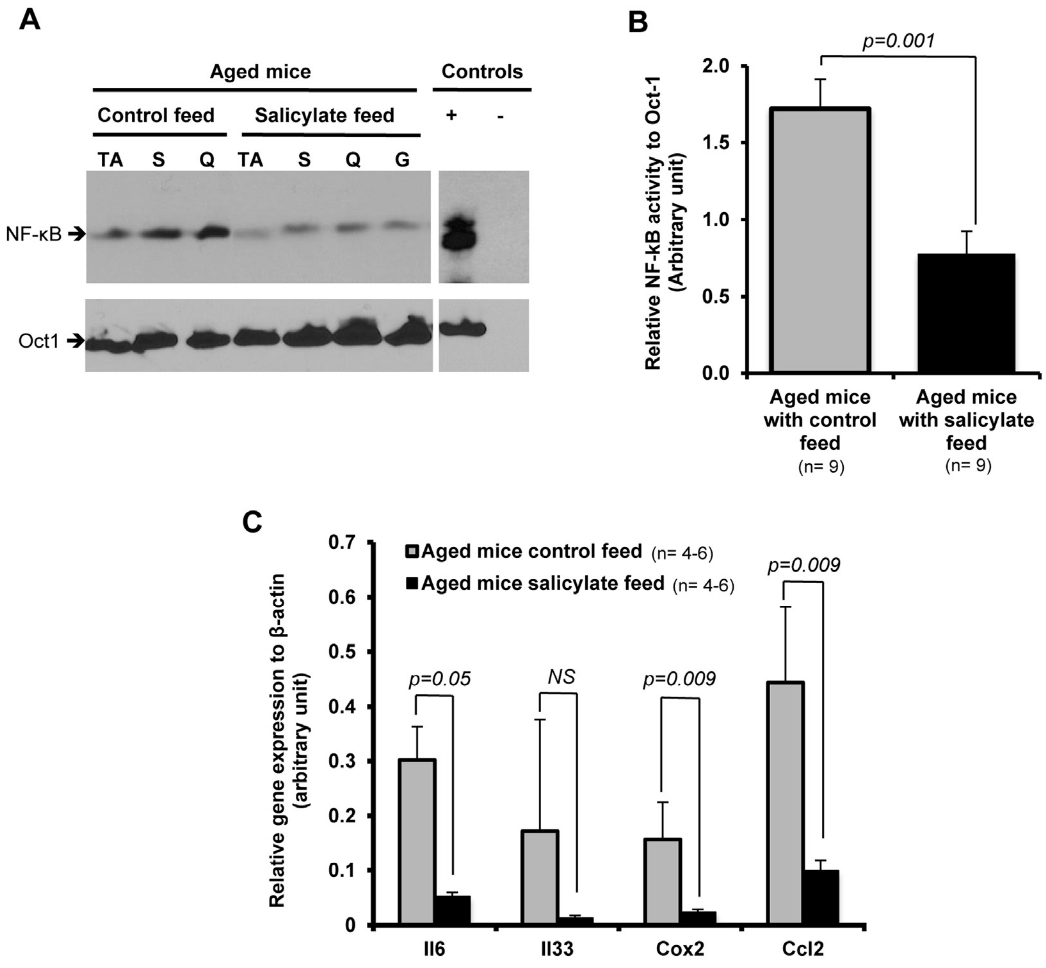


**Figure S8. Sodium salicylate treatment in aged mice reduces NF-κB activity in muscle and satellite cells.** (**A**, **B**) EMSAs performed on whole hind limb muscles of aged mice with or without salicylate treatment demonstrated a 50% decrease in NF-κB activity with salicylate treatment. Oct1 was used as a loading control. (**C**) Satellite cells isolated from these muscles similarly displayed evidence of reduced NF-κB transcriptional activity measured by mRNA level of direct targets or activators of NF-κB. All mice were 24 months in age initially and then received control feed or sodium salicylate feed *ad libitum* for 6 weeks. All data plotted as mean ± s.e.m. Student's *t* test was used for statistical analysis. *NS*: not statistically significant.

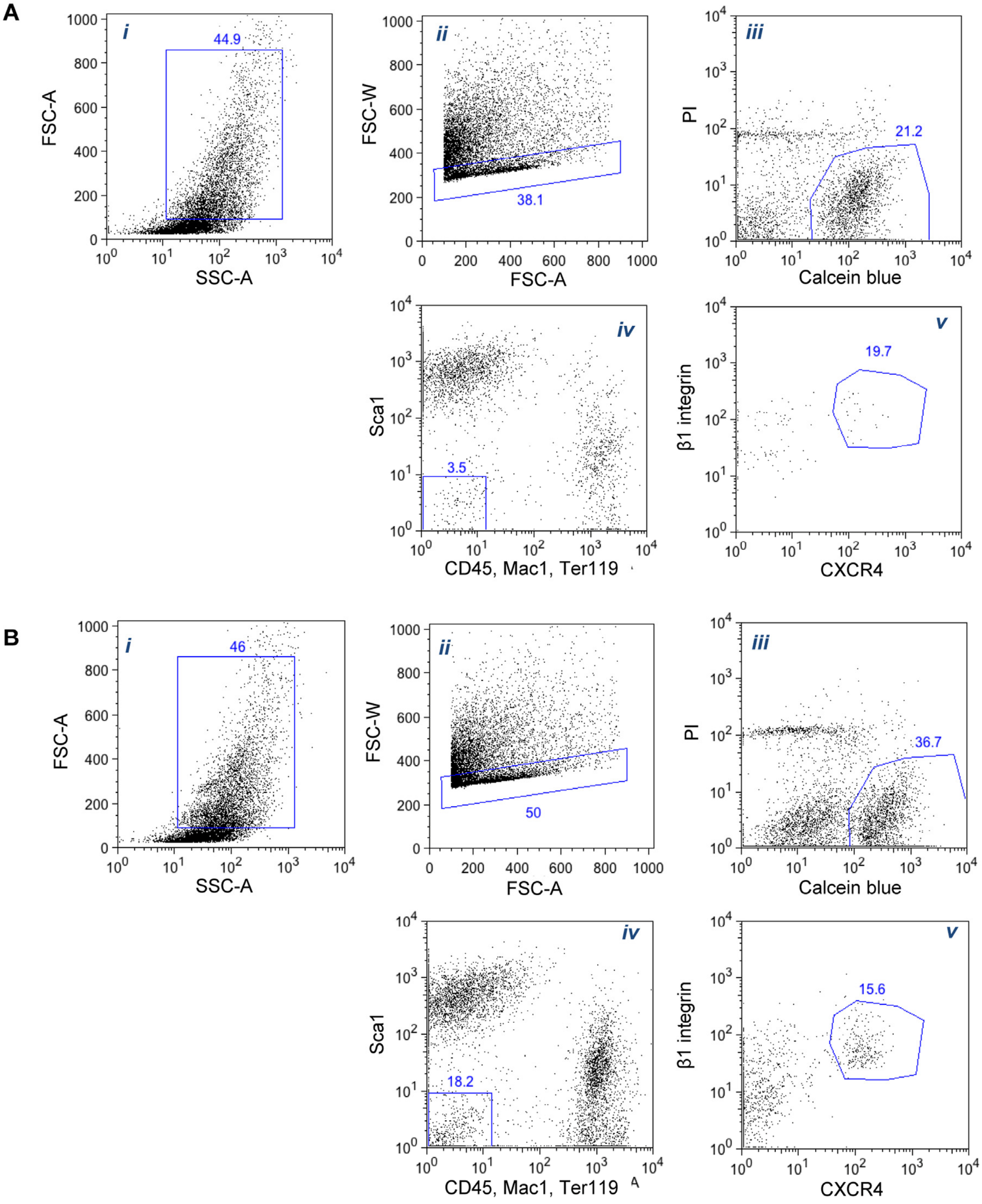


**Figure S9. Satellite cell frequency within the myofiber-associated cell compartment is not altered in aged mice treated with either control or sodium salicylate feed.** (**A**, **B**) Myofiber associated cell sorting utilizing flow cytometry was performed as previously described [4,21,42,43]. Representative flow plots depict the gating strategy *(i, ii, iii, iv, v)* used in control (**A**) and sodium salicylate treated (**B**) aged mice. All mice were 24 months in age initially and then received control feed or sodium salicylate feed *ad libitum* for 6 weeks.

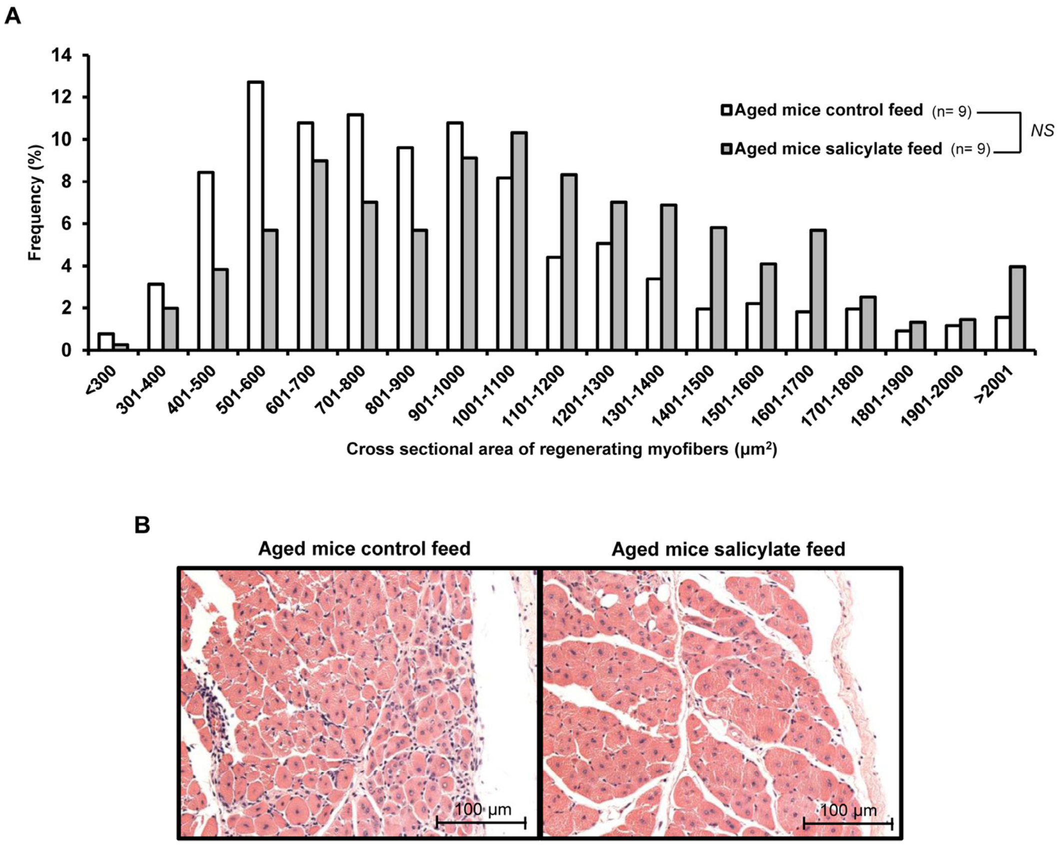


**Figure S10. Skeletal muscle regeneration in aged mice with or without sodium salicylate treatment at day 14 after injury.** (A, B) Aged mice with salicyate feed show a trend towards increased cross sectional areas of regenerating fibers compared to age-matched mice with control feed (n=6, $p$=0.27). All mice (22-24 month) received control feed or sodium salicylate feed *ad libitum* for 6-8 weeks prior to undergoing muscle harvest for dry ice injury. *NS*: not statistically significant.

A

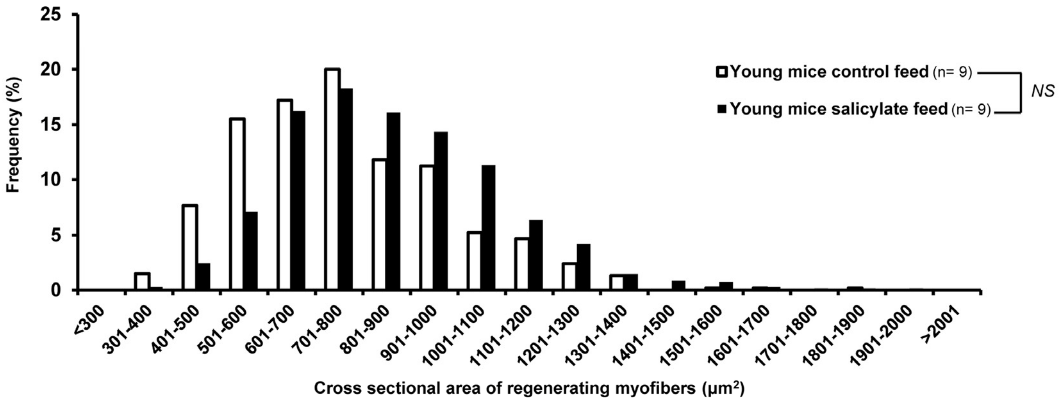


B

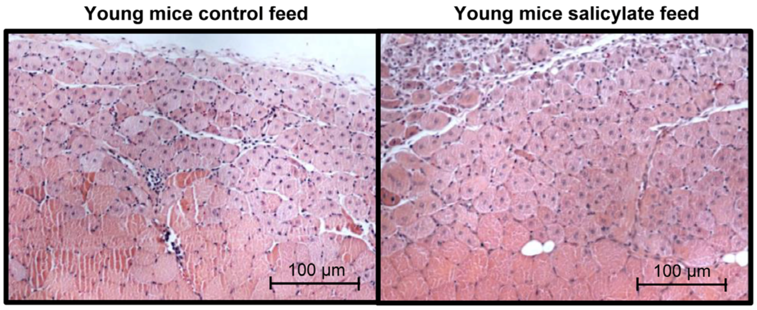


**Figure S11. Skeletal muscle regeneration in young mice with or without sodium salicylate treatment, analyzed at day 7 after injury.** (**A**) Cross-sectional areas of regenerating fibers in young mice are not statistically different ($p$=0.57) for animals receiving control or sodium salicylate feed (n=9). (**B**) Representative histological images are shown for the different treatment groups. Young mice ranged from 2-3 months of age. All mice received control feed or sodium salicylate feed *ad libitum* for 6-8 weeks prior to undergoing dry ice injury and subsequent muscle harvest for histology. Kruskal-Wallis test was used for statistical analysis in (A). *NS*: not statistically significant.

**Table S2. List of sequences of all primers utilized in qRT-PCR.**

| Gene | Forward primer | Reverse primer |
|---|---|---|
| *Actb* | 5'-TTCTGGTGCTTGTCTCACTGA-3' | 5'-CAGTATGTTCGGCTTCCCATTC-3' |
| *Il6* | 5'-GTTCTCTGGGAAATCGTGGA-3' | 5'-TCTGCAAGTTGCATCATCGTT-3' |
| *Il33* | 5'-TGCGTCTGTTGACACATTGA-3' | 5'-STGTACTCAGGGAGGCAGGA-3' |
| *Cox2* | 5'-TGAGCAACTATTCCAAACCAGC-3' | 5'-GCACGTAGTCTTCGATCACTATC-3' |
| *Ccl2* | 5'-AAGAGGATCACCAGCAGCAG-3' | 5'-TCTGGACCCATTCCTTCTTG-3' |
| *pla2g5* | 5'-CCAGGGGGCTTGCTAGAAC-'3 | 5'-AGCACCAATCAGTGCCATCC-'3 |

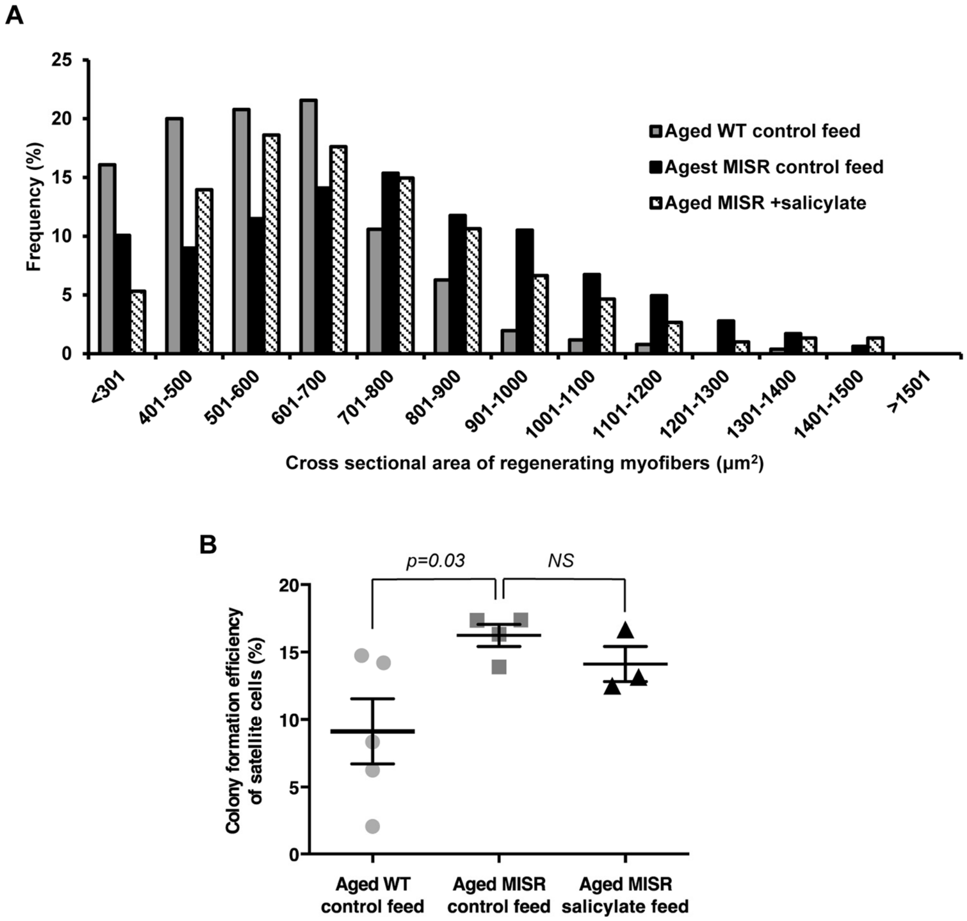


**Figure S12. Sodium salicylate treatment in aged MISR mice does not affect muscle regeneration after cryoinjury and does not alter myogenic activity of satellite cells.** (**A**) Cross-sectional areas of regenerating fibers after cyoinjury in aged MISR mice are not significantly changed in mice receiving sodium salicylate feed (n=3,4). (**B**) Colony formation efficiency of satellite cells isolated from aged MISR mice receiving control feed or sodium salicylate feed were not different. All mice received control feed or sodium salicylate feed *ad libitum* for 6-8 weeks prior to undergoing cryoinjury and subsequent muscle harvest for satellite cell isolation. Regardless of treatment, aged MISR mice showed improved muscle regeneration and myogenic activity of satellite cells as compared to aged WT mice (see Figure 2). Kruskal-Wallis test was used for statistical analysis in (**A**), and Student's *t* test in (**B**). *NS*: not statistically significant.

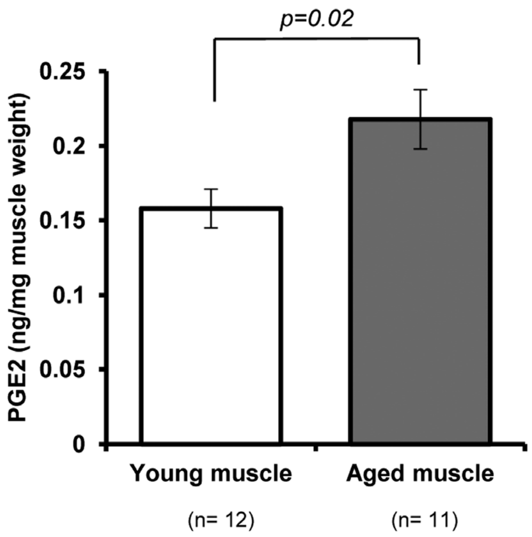


**Figure S13. Prostaglandin E2 (PGE2) levels in muscle increase with age.** ELISA detecting PGE2 demonstrates higher PGE2 levels in muscle extracts of old mice than those of young mice. All data plotted as mean ± s.e.m. Student's *t* test was used for statistical analysis.
